# Reconsidering the Mind-Wandering Reader: Predictive Processing, Probability Designs, and Enculturation

**DOI:** 10.3389/fpsyg.2018.02648

**Published:** 2019-01-08

**Authors:** Regina E. Fabry, Karin Kukkonen

**Affiliations:** ^1^Department of Philosophy II, Ruhr University Bochum, Bochum, Germany; ^2^Department of Literature, Area Studies and European Languages, University of Oslo, Oslo, Norway

**Keywords:** mind-wandering, reading, predictive processing, enculturated cognition, eye-tracking, literature

## Abstract

Studies on mind-wandering frequently use reading as an experimental task. In these studies, reading is conceived as a cognitive process that potentially offers a contrast to mind-wandering, because it seems to be task-related, goal-directed and stimulus-dependent. More recent work attempts to avoid the dichotomy of successful cognitive processes and processes of mind-wandering found in earlier studies. We approach the issue from the perspective that texts provoke modes of cognitive involvement different from the information processing and recall account that underlies many early mind-wandering studies. After all, reading itself is an umbrella term for activities that are characterized by a variety of phenomenological and functional signatures. We conceptualize reading and mind-wandering in relation to each other through the framework of enculturated predictive processing, which is informed by research in literary studies. Earlier accounts think of reading predominantly in terms of the construction of situation models that organize textual information. By contrast, enculturated predictive processing foregrounds shifting stances readers can take in relation to the text. Characters featuring in literary texts might be mind-wandering themselves, or passages in literary style might make the construction of a clearly defined situational model impossible. Furthermore, we take into account that texts often elicit mind-wandering through the construction of task-relevant and attention-driven virtual scenarios in readers' minds. This more complex account of reading can enrich recent attempts to understand mind-wandering as a complex, multi-dimensional phenomenon. The study of mind-wandering can benefit, we argue, from a closer attention to the process of reading and to the texts it deploys as stimuli. The emerging perspective from enculturated predictive processing and literary studies makes distinctions in reading that in turn enable research on mind-wandering to ask more precise questions about (1) different kinds of mind-wandering, (2) different modes of reading, and (3) how and where they interconnect across time.

## Introduction

The study of mind-wandering has time and again drawn on reading^1^. Deployed as a task-related, goal-directed and stimulus-dependent process, in many studies investigating mind-wandering, reading becomes the backdrop against which the onset of mind-wandering episodes is measured (Schooler et al., 2004, 2014; Smallwood et al., 2008; Reichle et al., 2010; Uzzaman and Joordens, 2011; Varao Sousa et al., 2013; Broadway et al., 2015; Sanders et al., 2017). More recently, however, research has moved toward an investigation of the multiple components and the dynamical unfolding of mind-wandering (Christoff et al., 2016). In line with this more dynamical perspective on mind-wandering, we suggest that reading, a cultural practice that comes in a variety of modes, should not be used unproblematically as a control task in experiments on mind-wandering. In turn, if research on mind-wandering pays closer attention to the complexity of reading, we propose, this might also support attempts to capture the complexity and dynamics of mind-wandering. We survey the state of research on mind-wandering featuring reading tasks, with particular attention to reading tasks involving literary texts, since these help us outline the underlying assumptions about reading on which these studies depend. A look at reading from the perspective of literary studies, empirical research on reading, and philosophy reveals, however, that reading is by far not always a unified task and that mind-wandering itself might be necessary for productive reading (certainly where literary texts are concerned). After reviewing the current state of research on mind-wandering and literary reading (State of research: Mind-wandering and reading), we reconceptualise the relationship between mind-wandering and reading through the approach of enculturated predictive processing (Enculturated predictive processing and the active exploitation of probability designs). In particular, we connect mind-wandering with processes of virtual exploration (epistemic active inference) of narrative and linguistic dimensions of literary texts (A new perspective on the relation of mind-wandering and reading) From this perspective, we develop practical implications and outline how a more fine-grained understanding of reading can contribute to the empirical study of mind-wandering (Concluding Remarks and Outlook).

## State of Research: Mind-Wandering and Reading

For a long time, researchers in the cognitive sciences were mostly interested in participants paying close attention to the cognitive tasks (e.g., reading, problem solving, reasoning) they are required to complete under laboratory and every-day conditions. The relevant on-going cognitive processes are therefore supposed to be task-related (Smallwood, 2011; Broadway et al., 2015; Metzinger, 2015; Smallwood and Schooler, 2015; Irving, 2016), goal-directed (Mooneyham and Schooler, 2013; Irving, 2016), and stimulus-dependent (Schooler et al., 2011; Broadway et al., 2015; Smallwood and Schooler, 2015; Konishi and Smallwood, 2016)^2^. These tacit assumptions with regard to function are often combined with the phenomenological postulate that the phenomenal experiences of human cognitive systems are completely anchored in the here and now of the completion of a cognitive task (Metzinger, 2013). Since functional magnetic resonance imaging (fMRI) studies (Shulman et al., 1997; Raichle et al., 2001) have shown that there is a cortical network that is reliably and consistently activated during resting states, these functional and phenomenological assumptions have increasingly been called into doubt (Christoff et al., 2016). This network, which is now called the *default mode network* (DMN), comprises regions in the posterior cingulate cortex and the medial prefrontal cortex (Fox and Christoff, 2014; Broadway et al., 2015; Smallwood and Schooler, 2015). Other contributing regions include the lateral prefrontal cortex, the medial and lateral parietal cortices, and parts of the temporal lobe (Schooler et al., 2011; Dixon et al., 2014; Christoff et al., 2016). Activations in parts of the DMN are associated with *mind-wandering* (see Andrews-Hanna et al., 2010). It should be noted at the outset that this network is “heterogeneous in such a way that different parts of the DMN are selective to specific tasks and types of processing” including processes that would be classified as mind-wandering and a range of other cognitive activities (Axelrod et al., 2017, p. 905). This dynamicity of activations of the DMN has led to the proposal to divide it into functional sub-components that are flexibly coupled to other nodes in the network and cerebral areas that are not part of the DMN (Christoff et al., 2016). Very recently, this view on the neuronal realization of mind-wandering has led to the methodological proposal to depict mind-wandering as an umbrella term for partly distinct types of mind-wandering, which are connected to each other by “family resemblances,” rather than depicting it as a homogenous phenomenon without any variations (Seli et al., 2018).

These latest developments take a step away from the shared assumption made by researchers who have used reading tasks in earlier studies as a backdrop against which the properties of mind-wandering have been investigated.

In most of the mind-wandering literature, the relationship between those whose minds are wandering and their situational context serves the conceptual specification of the properties of mind-wandering episodes. These properties can vary along different dimensions, namely task-relatedness/task-unrelatedness, goal-directedness/goal-undirectedness, and stimulus-dependence/stimulus-independence. In addition, Dixon et al. (2014) have suggested to distinguish between externally and internally directed cognitive processes. On their view, task-related cognition would be a case of “externally directed cognition” that is characterized by the attention trained on (parts of) the local environment. By contrast, “internally directed cognition” comprises attention aiming at processes that are classically construed as being detached from the local environment and being “internal” to human cognitive systems, e.g., remembering, imagining, and relevant to our present purposes, mind-wandering. We will see later on that this internal-external distinction is theoretically problematic for a number of reasons. For the moment, it is important to note that this distinction is frequently employed by some, but not all researchers who seek to explain mind-wandering (for examples, see Smallwood et al., 2007a; Smallwood, 2011; Schooler et al., 2014; Smallwood and Schooler, 2015). It has also been argued that mind-wandering is characterized by the decoupling of attention from current task demands in the “external” local environment (Smallwood, 2011; Broadway et al., 2015; Smallwood and Schooler, 2015; Konishi and Smallwood, 2016; Sanders et al., 2017). For example, Smallwood (2011) argues that “when internal thoughts and feelings form our train of thought, attention is disengaged or *decoupled* from the external world” (p., 64; italics in original).

As the metaphor of “mind-wandering” already suggests, the phenomenology of mind-wandering is characterized by experiences that are detached from current task demands.

Reading has turned out to be a paradigm task for the empirical investigation of the impact of mind-wandering on cognitive performance. The reason is that reading comprehension is a well-researched cognitive process that can be reliably operationalised and controlled (Snowling and Hulme, 2007; McNamara and Magliano, 2009). Reading comprehension can be tested conveniently when one asks participants to complete questionnaires about the content of the reading material. Furthermore, the temporal progression of the reading process and the requirement of sustained attention in reading tasks appear to be highly suitable for the systematic investigation of mind-wandering that also unfolds through time. The presented reading materials vary across different studies and range from the entirety of Jane Austen's, 2008/1811 novel *Sense and Sensibility* (Austen's, 2008; Reichle et al., 2010) and an edited version of Conan Doyle's, 2011/1892 short story *The Red-Headed League* (Smallwood et al., 2008; Conan Doyle's, 2011; Franklin et al., 2011) to an excerpt from Lev Tolstoy's, 2010/1869 novel *War and Peace* (Schooler et al., 2004; Tolstoy's, 2010; Uzzaman and Joordens, 2011) and excerpts from Bryson's (2003) popular fiction book *A Short History of Nearly Everything* (Varao Sousa et al., 2013; Broadway et al., 2015; Sanders et al., 2017). Interestingly, none of the studies cited above provides reasons for the choice of the employed text material.

As we will see in the course of the present paper, however, the specific properties of the text material have an influence on the timing and duration of cognitive episodes classified as mind-wandering. Indeed, studies on reading and mind-wandering in instructional and expository texts take the complexity of the text into account (Feng et al., 2013; Forrin et al., 2017; Mills et al., 2017). However, it should be noted that Feng et al. (2013) control for text difficulty by operationalising it in terms of syntactic complexity and word frequency (without mentioning any details about the assessment of these properties). Both Mills et al. (2017) and Forrin et al. (2017) determine text complexity by using the Kincaid Grade Level scale, which measures the difficulty of texts relative to school grades. Interestingly, Mills et al. (2017) complement the Kincaid Grade Level scale with a measurement of the concreteness of the words occurring in the reading material. These are important, yet incomplete steps toward a better understanding of text complexity. Certainly, such textual complexity should also be taken into account when working with texts which are considered the most complex of all — literature. It is also worth mentioning that Schooler et al. (2004) acknowledge that “breaks in the text” and “perhaps even textual conventions (e.g., boldface or italicized text)” might have a positive impact on the ability to terminate mind-wandering episodes as a result of “a moment of metaconscious scrutiny” (p. 221). Furthermore, these researchers leave room for the possibility that short mind-wandering episodes might actually lead to increased reading comprehension performance in some cases. These cases are supposed to be partly determined by the textual properties of the reading material. We will come back to these conjectures at the end of this section.

In general, mind-wandering episodes are identified based on participants' self-reports, which are either probe-caught (Smallwood et al., 2008; Franklin et al., 2011; Uzzaman and Joordens, 2011; Varao Sousa et al., 2013; Broadway et al., 2015), self-caught (Sanders et al., 2017), or a combination of both (Schooler et al., 2004; Reichle et al., 2010). In cases of *probe-caught* self-reports, participants are systematically asked by the experimenter whether they were mind-wandering immediately prior to the probe. In cases of *self-caught* reports, participants are instructed to make a brief report to the experimenter whenever they have caught themselves mind-wandering. Both types of self-reports are motivated by the idea that participants can retrospectively identify mind-wandering episodes once they have regained *meta-awareness* of their cognitive processes (Smallwood et al., 2007b; Schooler et al., 2011)^3^.

Research on the relationship between reading comprehension and mind-wandering has led previous research to posit a distinction between two types of mind-wandering: tuning out and zoning out (see Schooler et al., 2004, 2011; Smallwood et al., 2007b; Dixon et al., 2014; Metzinger, 2015, 2018; Smallwood and Schooler, 2015). When readers *tune out*, they can become aware that they are mind-wandering and, while they are doing it, might be able to control their train of thought at least to some degree. By contrast, in cases of *zoning out*, readers are assumed to be unaware that they are mind-wandering. Nevertheless, they will recognize that they were mind-wandering when they come to the end of the mind-wandering episode and will be able to report its phenomenal content retrospectively.^4^

The results of the afore-mentioned studies indicate that mind-wandering is reliably and consistently associated with poor reading comprehension (Schooler et al., 2004; Franklin et al., 2011; Broadway et al., 2015; Sanders et al., 2017; see also Schooler et al., 2014; Smallwood and Schooler, 2015). The effect of mind-wandering episodes has been observed at word, sentence and text levels. Mind-wandering seems to impede readers' ability to draw inferences about the content of Conan-Doyle's *The Red-Headed League* (Smallwood et al., 2008). Part of the reason for this finding could be that the text is not processed deeply during mind-wandering episodes (Uzzaman and Joordens, 2011). In addition to lacking inferential coherence, it has also been suggested that mind-wandering readers show decreased sensitivity to the lexical properties of the text (Reichle et al., 2010; Franklin et al., 2011). Analyses of eye movement patterns during mind-wandering episodes show this: When compared to attentive reading, reading during mind-wandering episodes is associated with fewer fixations, longer fixation durations, and fewer regressions (Reichle et al., 2010; Smilek et al., 2010; Uzzaman and Joordens, 2011). The frequency and predictability of words in a semantic and syntactic context, it is assumed in reading studies, have a strong influence on the duration and location of fixations and the probability of word skipping (Rayner, 1998; Drieghe et al., 2004; Kliegl et al., 2004). Highly frequent and predictable words in a given context are likely to be either skipped or fixated for a shorter period of time in comparison to words of average or low frequency and predictability. Any attention-guided reading process would be automatically structured by such effects of frequency and predictability. During mind-wandering episodes they therefore appear to be absent (Schooler et al., 2014).

Different kinds of reading, however, yield different results. The activities of reading silently, reading out loud and listening to a text each feature different functional contributions of motor patterns to the reading process and they are likely to support different “depths” of engagement. Taking findings from the eye-tracking literature as their starting point, Varao Sousa et al. (2013) explore whether a greater or smaller degree of involvement of motor patterns in reading makes it more likely that readers start to mind-wander. Varao Sousa et al.'s (2013) study indicates that the frequency of mind-wandering episodes is the highest for the listening condition, followed by silent reading and reading aloud. The researchers conclude that their study “broadly supports the notion that a more physically engaged reading experience means readers are likely to spend less time mind wandering” (Varao Sousa et al., 2013, p. 3).

Smallwood (2011) has developed a model that seeks to provide an explanation for how mind-wandering relates to reading comprehension, based on empirical findings and existing accounts of reading comprehension (McNamara and Magliano, 2009). In line with existing computational and representational three-stage models of reading comprehension (Kintsch and van Dijk, 1978; van Dijk and Kintsch, 1983; Zwaan and Radvansky, 1998), Smallwood assumes that reading comprehension is constituted by lexical, propositional, and situation-model levels. Representations of the reading material at these different levels are subject to both bottom-up and top-down-influences. For example, what readers think the sentence “says” (i.e., its propositional representations) will be influenced by the representation of the words deployed (i.e., lexical representations) at the level below. At the same time, propositional representations are influenced by what readers think is the state of affairs in the narrative (i.e., the situation model) at the level above, which aims at a coherent representation of the narrative as a whole.

Smallwood's (2011) account of mind-wandering and reading comprehension relies on the internal-external distinction mentioned above. Drawing on the distinction between internally and externally directed attention, Smallwood (2011) explains the differences between attentive reading and mind-wandering during reading in the following terms (p. 72):

In normal reading, directed attention to the words on the page lead bottom-up processes and top-down feedback interactions to create and maintain a state of coupling between the internal state and the external representation of the narrative. During mindless reading, this interaction breaks down because the internal state of the reader and the external representation of the narrative become decoupled. This decoupling prevents the individual processing [of] the lexical features of the words on the page, impairs the formation of a propositional model of what was read and, ultimately, has implications for how effectively individuals can build a coherent situational model of what is read.

At each of the three representational levels (text, proposition, and situation model), attentional decoupling has a detrimental effect. The question arises, however, whether reading does not continue at a certain level while other levels detach. One might continue following the sequence of words on the page and remain attentionally coupled at the level of lexical representations, but not integrate these words into propositions, and thus become attentionally decoupled on the level of propositions or the situation model. We propose a different understanding of reading comprehension is necessary to account for the delicate relationships between readers and texts in the next section.

Mind-wandering in the case of reading has been characterized by task-unrelatedness, the absence of goal-directedness, stimulus-independence, unguided attention, and inward directedness. On this view, mind-wandering is detrimental to successful reading performance (Smallwood et al., 2007a; Smallwood, 2011; Mooneyham and Schooler, 2013). The assumptions are nicely captured by Fox and Christoff (2014): “In contrast to the more desirable pursuit of ‘rational’ thought, MW [i.e., mind-wandering] is often portrayed as undesirable–a wasteful mental diversion and potentially dangerous distraction” (p. 299). More recently, however, it has been acknowledged that mind-wandering might be beneficial for future planning, dishabituation during learning tasks, creative problem solving, and relief from boredom (Mooneyham and Schooler, 2013; Schooler et al., 2014). The general tenor is one of worry, though. If you let your mind wander, a recent article in *Current Directions in Psychological Science* warns, you will not be able to retain the content of what you read as well as is required in our information society (Szpunar, 2017).

As we have already noted, the mind-wandering literature is relatively reticent with comments on the properties of the reading process. Based on the studies on relationship between mind-wandering and reading we have reviewed above, one might get the impression that reading is simply understood as the opposite of mind-wandering: on task, goal-directed and stimulus-dependent. There are many cases, however, in which reading does not have all these properties, which are worth considering in more detail. How often do we read a newspaper article with the intention to retain its main points in memory? How often are we just curious to check what it is about before we completely forget about it? Is our goal when we pick up Jane Austen's (2008/1811) *Sense and Sensibility* to mine it for whatever information it holds about Regency Britain? Are we actually interested in tracing the intricacies of Austen's plot? Aren't readers rather looking for a kind of flow-state in literary reading that resembles mind-wandering in that readers detach their attention from the here and now?

Reading can be task-oriented, for example, when we read for information. However, even then we can skim the pages without attending properly to the lexical details. Readers can attend to language, the luxuriousness of Thomas Mann's prose, for example, without taking into account what it is they are reading about. If mind-wandering is connected to directing less attention to the lexical properties of the text, as the eye-tracking studies indicate (Reichle et al., 2010; Franklin et al., 2011), then the question arises whether there are linguistic regularities to the moments when readers' minds begin to wander. Does the prose tend to be particularly complex (and therefore demand too much attention)? Or does it tend to be too simple (and thereby leave readers to go off mentally on their own)? The studies that use eye-tracking for the identification of mind-wandering episodes have actually little to say about the particularities of the “lexical details” of the texts^5^. Indeed, one could argue that many texts are not designed to fulfill the criteria for reading in these mind-wandering studies. For example, the ambiguities of free indirect discourse and characters overhearing one another while pretending to busy themselves with something else in *Sense and Sensibility* are arguably not meant to be resolved while reading. Beyond the typographic conventions that Schooler et al. (2004) mention as introducing ”metacognitive scrutiny” (p. 221), however, also literary techniques that shape language could be considered as heightening meta-cognitive involvements (Kukkonen, Forthcoming b) and they could thereby not only terminate mind-wandering, as Schooler et al. (2004) have it, but shape it productively.

Let us take an example from Thomas Mann's, 2014/1954 novel *Bekenntnisse des Hochstaplers Felix Krull* (*Confessions of Felix Krull*; Mann's, 2014). When Krull visits the circus, he admires the skill and disdain for death of the trapeze artiste Andromache. Even after her performance he thinks of her “Andromache! Painful at once and elevating rested her being in my mind, when her performance had long ended and others had stepped into her place” (in the German: “Andromache! Schmerzlich zugleich und erhebend lag mir ihr Wesen im Sinn, als längst ihre Nummer vorüber und anderes an ihre Stelle getreten war”; p. 200)^6^. Krull attempts to follow the next performance, featuring horses, but he immediately thinks of her again. “A gorgeous sight, but I thought of Andromache. Splendid animal bodies, and between animal and angel, I mused, stands man. Closer to the animal he stands, this much we have to admit. She however, my adored, even though she was body through and through, but a body more chaste, excluded from the human, stood much further with the angels” (“Ein prächtiger Anblick, aber ich gedachte Andromaches. Herrliche Tierleiber, und zwischen Tier und Engel, so sann ich, stehet der Mensch. Näher zum Tiere stehet er, das wollen wir einräumen, Sie aber, meine Angebetete, obgleich Leib ganz und gar, aber keuscher, vom Menschlichen ausgeschlossener Leib, stand viel weiter hin zu den Engeln” p. 201). Krull continually goes back from the current events in the circus to the inner image of Andromache. As Mann's prose repeats the verb “stehen” several times, echoing the noun “Stelle” in the first sentence, he shifts it between the literal, embodied meaning of “standing” in the arena of the circus and the abstract meaning of “standing” in relation to one another in Krull's imagined great chain of being. It is difficult to say how a situation model would be constructed here, since the spatial information we get from the verb is not reliable but oscillates between what takes place in the fictional world and what Krull imagines. As the embodied verb shifts between the present and the imagined, mind-wandering might be facilitated (this by itself is an assumption open to empirical investigation, of course). And such mind-wandering would not be decoupled from the rest of the text but shaped by the predictive frame generated through the well-known notion of a great chain of being along which Krull's thoughts make sense.

Thus mind-wandering can be represented in the texts deployed to study that phenomenon. This issue, which is most obvious for literary narratives, is not addressed by studies that use reading as a task to investigate mind-wandering. Marianne in *Sense and Sensibility* is swept off her feet by the dashing Willoughby and, moreover, by her imagination and effusive sensibility. Felix Krull does not only contemplate the great chain of being in the circus, he is also carried into admiring the complexities of the long history of the earth on a train-ride to Lisbon, as the director of the museum of natural history lays it out for him. It gets really difficult to determine what it means to be “on task” here. As Krull recounts this spectacular lecture, he does not provide readers with much specific information but rather with a feeling of the experience widening his horizons. Here as in the earlier example, the issue of the indeterminacy on the lexical level moves across to higher levels of meaning-making. What is the relevant situation model here? Krull listens to Professor Kuckuck's lecture? Natural history has a spectacularly long range? Arguably, readers come to understand something about Krull as a character and about the range and ambition of Mann's novel, which can only be achieved through mind-wandering on the part of readers themselves. If readers mind-wander here, for example, when the embodied verb shifts in reference from the train compartment to the cosmos, they do not necessarily decouple their attention here but rather follow the design of the literary text. Moreover, as we have seen with the Andromache passage, Mann introduces several passages that work according to this pattern. As readers move through the text, we could say, the text scaffolds their mind-wandering. Time and again, these mind-wandering passages expand the reach of Mann's narrative.

Reading is clearly stimulus-dependent in that it is based on a written text. However, what brings texts to life when reading often looks very much like mind-wandering. Reader-response criticism in literary theory, for example, draws on hermeneutics and phenomenology when it argues for a reader's share in filling the blanks which written texts inevitably leave (Iser, 1972, 1984). Readers' imagination supplies the material for this. Indeed, the Romantic notion of the imagination has been discussed as a conceptualization *avant la lettre* of the processes associated with activations in the default-mode network (Richardson, 2011). Not only the poetry of the Romantics, but literary writing more generally, has been understood as eliciting mental imagery that shapes readers' deep understanding of these texts (Scarry, 1995; Kuzmičová, 2014; Troscianko, 2014). The flow state of literary reading has also been understood to facilitate explorative thoughts on the part of readers, where they configure and reconfigure the meaning of the text in multiple ways (Kukkonen, Forthcoming b). Readers retain a certain level of spontaneous thought, which has been related to mind-wandering (Christoff et al., 2016), rather than operate completely depending on the text. This has been most clearly argued for the case of literary fiction. However, “tuning out” (where readers remain aware that they are mind-wandering) has also been discussed for experiments with non-fictional texts.

If reading indeed were primarily a task that is goal-driven and stimulus-dependent, one would expect that re-reading would lead to a much higher degree of mind-wandering because the information to be retained from the text is already known. Phillips et al. (2016), to our knowledge the only study on mind-wandering and reading where readers were asked to read the same text twice, confirms this idea. They report that re-reading leads to faster reading, no improvement in recall and significantly increased intentional mind-wandering. This effect, however, might be due to the possibly unrewarding nature of the reading material in this experiment (an electronic textbook on “critical thinking and scientific reasoning” that accompanies a computerized learning game). It is a hypothesis open to future empirical investigation that the case is different for reading literary texts. Readers can take up Austen's (2008/1811) *Sense and Sensibility* a second time and read with just as much attention as the first time. It seems that this phenomenon even applies to genres like detective fiction, where the narrative is much more obviously information-based than in Austen. The fact that readers have read before the revelation who is the killer or the resolution to a suspenseful passage of the narrative, however, does not seem to diminish their engagement (see Gerrig, 1993 who calls the phenomenon “anomalous suspense”). There is clearly more at stake in reading than mere “comprehension” and drawing inferences about the content of detective stories. This line of reasoning points to an intriguing empirical question that underscores the differences between literary and expository texts when it comes to mind-wandering: Does the mind wander more or less when the *literary* text is known?

A number of further empirical questions arise from these observations around Felix Krull's distracted mind and our previous considerations on literary reading. First, do passages in which characters and narrators mind-wander facilitate mind-wandering in readers? Is there a correspondence between what we could call first-order mind-wandering (represented in fiction) and second-order mind-wandering (elicited by fiction)? Second, do shifts in embodied language, for example between interoception and exteroception, between bodies present in the fictional world or imagined by the characters, facilitate mind-wandering? And, third, does controlled mind-wandering get entrained as we read a text? The assumption here would be that the link between first-order and second-order mind-wandering manifests itself more and more in terms of tuning out (that is, mind-wandering while you are aware of it) and less and less in terms of zoning out (that is, mind-wandering while you are not aware of it) as readers continue in the narrative. In tuning out, readers can actively shape the mind-wandering episodes; in zoning out, however, there is no active exchange between the textual design and readers' cognitive processes. It is important to note that mind-wandering is a dynamical process that unfolds across time. While a lack of intentionality and meta-awareness can be a signature of the beginning of mind-wandering episodes, the degree of intentionality and meta-awareness can diverge in the course of the episode (Seli et al., 2017). The distinction between tuning out and zoning out refers to the degree of meta-awareness that is involved in a certain mind-wandering episode. It is possible that both tuning out and zoning out are characterized by the presence of some degree of intentionality, such that meta-awareness and intentionality are distinct dimensions along which the unfolding of mind-wandering episodes can vary.

A few studies on mind-wandering and reading have taken into account different modes of approaching the text. Varao Sousa et al. (2013), as we have seen, found that for reading comprehension and recall, the main criteria of “successful reading,” reading out loud is a better strategy than silent reading. It would be an open question, however, whether texts that depend on mind-wandering on the part of readers, such as *Confessions of Felix Krull*, would be as well-served by reading out loud as are the chapters from Bryson's (2003) *Short History of Nearly Everything*, which has been much used in these studies (Broadway et al., 2015; Sanders et al., 2017). (Indeed, the ways in which Krull associates knowledge to his own experience, are arguably at stake in Bryson, too, Mann seems to imply that the only way to experience a text like Bryson's properly is through mind-wandering.) Insofar as reading is the gold standard of cognitive performance in mind-wandering studies, it is striking how often reading is taken for granted, while discussions of reading comprehension argue for the need to develop more complex and comprehensive models (McNamara and Magliano, 2009). In the next section, we will suggest to explore reading from the perspective of enculturated predictive processing with a particular emphasis on the probability designs that describe the relationship between the properties of texts and the unfolding of narratives and the activity of reading. In a second step, we will show how this approach can enrich our understanding of reading, mind-wandering, and their intricate relationship.

## Enculturated Predictive Processing and the Active Exploitation of Probability Designs

Research on reading comprehension has articulated the need to attend to the specifics of the text, on the one hand, and to the skills and capacities of the reader on the other hand (see McNamara and Magliano, 2009), rather than rely on the representation of situation models alone, as it underlies much of the mind-wandering research drawing on reading. We propose *enculturated predictive processing* as a theoretical model that answers this call. If we define cognitive practices as socio-culturally distributed and embodied interactions with cognitive tools, then reading becomes a paradigm case of such practices involving symbolic linguistic representations (Menary, 2007, 2012, 2015; Fabry, 2015, 2018). Reading is socio-culturally distributed, because it is a normatively constrained “patterned practice” that is shared by many members of a socio-cultural community (Roepstorff et al., 2010). Furthermore, reading is embodied, because it is dependent upon the bodily manipulation of symbols (Rowlands, 1999; Menary, 2007) and resonances of the embodied dimensions of words and syntax (Kukkonen, 2014b).

Competence in reading and other cognitive practices is the result of *enculturation*. One can approach enculturation on the personal level (by considering explicit training and instruction in reading) and on the sub-personal level (by considering readers' brain-based and ocular-motor processes going on as they learn to read). On a personal level of analysis, enculturation is realized by cultural learning. Cultural learning is a specific variant of social learning, which transmits culturally evolved skills and knowledge from one generation to the next (Heyes, 2012, 2016; Henrich, 2016). On a sub-personal level of analysis, enculturation is realized by learning driven plasticity (LDP) and learning driven bodily adaptability (LDBA). The acquisition of reading and other cognitive practices is associated with plastic changes to the structural, functional, and effective connectivity of relevant brain regions (Menary, 2015; Fabry, 2018), for example the left ventral occipito-temporal (vOT) region (McCandliss et al., 2003; Price and Devlin, 2003; Dehaene, 2010), the left inferior frontal gyrus (Turkeltaub et al., 2003; Carreiras et al., 2014), and the left superior temporal gyrus (Braze et al., 2011; Brennan and Pylkkänen, 2012).

Enculturation is also dependent upon the development and refinement of motor patterns that realize the bodily manipulation of symbolic linguistic representations and other cognitive tools. In the case of reading acquisition, LDBA is associated with the development of eye movement patterns that allow readers to bodily manipulate texts efficiently and effectively. Contrasts of novice and proficient readers indicate that proficient reading is characterized by a decrease of fixation durations and re-fixations, as well as an increase of saccadic amplitudes (Huestegge et al., 2009; Joseph and Liversedge, 2013; Seassau et al., 2013).

If reading competence is the result of enculturation, then how does it play out in the reading process itself? The account of enculturation, as it has been developed so far, leaves partly unexplained how exactly proficient and expert reading is realized in the brain and the rest of the body. We propose that the theoretical integration of the enculturation account with predictive processing provides us with important conceptual tools that help close this gap.

According to recently developed *predictive processing* accounts, perceptual, active, and cognitive phenomena are realized by the minimization of the discrepancies between top-down predictions and bottom-up prediction error signals (Friston, 2005, 2010; Clark, 2013, 2016; Hohwy, 2013). The assumption is that the human cortex implements a hierarchical generative model. Each layer of the hierarchy is causally connected to the adjacent layers above and below. Each layer generates predictions of the most probable bottom-up signal from the layer below. The divergence of the top-down prediction and the bottom-up signal from the layer below is the prediction error. The prediction error serves as the bottom-up signal to the next layer above. The objective of this iterative process, which is realized at multiple levels of the hierarchical generative model, is the minimization of prediction error (Hohwy, 2011, 2013; Clark, 2013, 2016). The concurrent updating of the hierarchical generative model as a function of prediction error minimization is assumed to approximate Bayesian belief-updating in the light of new evidence (Friston, 2010; Clark, 2013; Hohwy, 2013, 2015).

For a concrete example, consider again the passage from *Felix Krull*.“Ein prächtiger Anblick, aber ich gedachte Andromaches.” When reading this sentence, readers fluent in German will expect certain letter sequences and divergences offer prediction errors. “Präschtig” would be a prediction error at the level of letter recognition that could be explained through new predictions at higher levels, such as a typo or a misspelling reflecting how those who speak German with a Hessian accent would pronounce “prächtig.” At the level of morphological and syntactical predictions, readers predict possible continuations for different components of a word and different words in a sentence segment. “Ein prächtiger…” leads readers to expect a noun within a certain semantic range, and “ich gedachte” leads readers to expect a noun in the genitive (“Andromaches”) or in the accusative if followed by an infinitive (“Andromache wieder aufzusuchen”). These predictions on the level of spelling, syntax and semantics are not formed and applied by the reader as a whole, but are constantly generated and updated sub-personally by the hierarchical generative model. These concurrent predictions are at play in any mode of reading, not just literary texts like *Felix Krull*.

There are two complementary ways to minimize prediction error, and we shall see how they relate to the cognitive processes involved in reading and mind-wandering as discussed above. In *perceptual inference*, top-down predictions are updated as a result of prediction error. In *active inference*, embodied actions change the available input to bring about predicted sensory states (Friston, 2010; Clark, 2013, 2016; Hohwy, 2013; Seth, 2015b). Eye movements are an especially important type of active inference (Friston et al., 2012) and more particularly of epistemic active inference (Seth, 2015a; Seth and Friston, 2016). In contrast to instrumental active inference, whose main purpose is to actively control variables in the sensory signals, *epistemic active inference* is defined as the process of “revealing the causes of sensory signals” (Seth, 2015a, p. 7). Roaming an environment with your gaze would be an example of epistemic active inference. However, epistemic active inference does not necessarily lead to the execution of overt motor actions in all cases. The class of epistemic active inference also includes cases of “the retrieval (or construction) of episodic memories through internal rehearsal (or simulation)” (Pezzulo et al., 2016, p. 323). For the sake of conceptual clarity, we make a distinction between *palpable* epistemic active inference (e.g., eye movements) and *virtual* epistemic active inference (e.g., the generation of virtual scenarios).

Whatever perceptual and active inferences contribute to the reduction of prediction errors is modulated by the estimated *precision* (i.e., the inverse variance) of prediction error signals (Hohwy, 2013; Clark, 2015; Seth, 2015b). It has been suggested that precision estimation is equivalent to attention (Feldman and Friston, 2010; Hohwy, 2013; Clark, 2016). Eye movements, generating virtual scenarios, and attention are thus conceptually connected in predictive processing.

The emerging enculturated predictive processing framework, which integrates the enculturation account with predictive processing, provides us with revealing conceptual tools that help us interpret and contextualize empirical data (Fabry, 2017, 2018). It also leads to the development of new theoretical considerations on reading and—as we will see shortly—its relation to mind-wandering. According to this framework, predictive processing can make important contributions to our understanding of reading on a sub-personal level of analysis. First, we can relate what we know about the cerebral realization of reading to precision-modulated perceptual inference and thus improve our grasp on the role which lexical, syntactic, and semantic understanding plays in reading. Following Price and Devlin's (2011) predictive processing account of visual word recognition, we suggest that significant activation patterns in the left vOT area and connected temporal and inferior frontal areas in the left hemisphere represent systematic prediction error signals. According to Price and Devlin (2011), reading acquisition is a special case of prediction error minimization, which leads to more accurate top-down predictions generated especially in frontal and temporal regions as a function of progressive reading experiences. The accuracy of top-down predictions and the optimality of precision estimates increases in the course of reading acquisition and multiple reading experiences during adolescence and adulthood.

Furthermore, predictive processing can integrate multiple empirical findings on the influence of specific linguistic properties on the reading process at the sentence level. For example, behavioral and neuroscientific studies suggest that the repetition of a certain syntactic structure in consecutive sentences makes it easier to read a target sentence that shares this structure (Weber and Indefrey, 2009; Tooley and Traxler, 2010; Kim et al., 2013). This syntactic priming effect is associated with decreased levels of activation in left-lateralised temporal areas, e.g., in the left inferior and middle temporal gyri (Noppeney and Price, 2004). Interpreted in terms of predictive processing, the syntactic priming effect occurs because recent prediction error minimization associated with the priming sentence informs the selection of future predictions targeted at the visual presentation of the primed sentence. Updated predictions as a result of processing the priming sentence renders certain predictions about upcoming syntactic structures in the primed sentence more likely than others. Accordingly, their top-down influence is strengthened and competing predictions are canceled out. Other robust reading effects at the sentence level that can be readily explained along similar lines include the ambiguity resolution effect (Snijders et al., 2009; Fine et al., 2013) and the syntactic complexity effect (Keller et al., 2001; Michael et al., 2001; Constable et al., 2004).

Second, predictive processing can also provide us with the descriptive and explanatory resources to account for the ocular-motor component of reading. The increased efficiency and effectiveness of eye movement patterns that are associated with the transition from novice to proficient reading can be interpreted in terms of precision-modulated palpable active inference. On this view, reading would be a case of *active looking* rather than passive seeing (Dewey, 1896; Findlay and Gilchrist, 2003), which requires readers' ocular-motor interaction with the text in a flexible and context-sensitive fashion. In close interaction with perceptual inference, palpable active inference becomes more accurate and precise and is therefore an effective means to manipulate texts in a bodily fashion. This is empirically supported by the studies mentioned above suggesting that proficient reading is associated with shorter fixation durations, less re-fixations, and larger saccadic amplitudes in comparison to novice reading. Furthermore, the association of eye movements with active inference can accommodate a wealth of empirical findings on robust eye movement effects, e.g., the word frequency effect (Rayner and Raney, 1996; Drieghe et al., 2004; Kliegl et al., 2004) and the word predictability effect (Ashby et al., 2005; White et al., 2005; Rayner et al., 2011). These effects suggest that it becomes more likely that readers skip words, and that the duration of fixations become longer as a function of the frequency and predictability of a certain target word in a sentence context. In terms of predictive processing, words that are highly frequent or highly predictable in a certain syntactic context are associated with accurate predictions based on previous reading experiences. If words are highly predictable, then readers do not need to actively sample the visual stimulus array through palpable active inference, and this is evidenced by shorter fixation durations and a higher probability that words will be skipped. The word frequency and predictability effects support the idea that reading is realized by the precision-modulated interplay of perceptual and palpable active inference in a context-dependent fashion. It is important to note that the relevance of enculturated predictive processing for the study of reading does not stop at the description of this process at the sentence level. The engagement with entire narrative texts can also be described as the enculturated minimization of prediction error. The reader's engagement with narratives would lead to updates of the hierarchical generative model by establishing “high-level elements” (Clark, 2016, 286) or “priors or even hyperpriors, sets of expectations that shape perception and guide action” (Roepstorff, 2013, p. 225). These (hyper-) priors would causally influence future reading experiences.

When readers engage with the texts commonly used in studies on mind-wandering, they not only work with the predictability and word frequency effects involved in word-recognition but also the development of the plot of their narratives and their style. New events in the plot of the narrative can be conceptualized as prediction errors that invite readers to revise their predictions on what is likely to happen next (Kukkonen, 2014a) and stylistic devices, as we have for example observed them in Mann's *The Confessions of Felix Krull*, shape readers' attention through shifts in precision and expectations about precision (Kukkonen, 2014b, Forthcoming b). Literary texts entail a “probability design” that provides carefully crafted prediction errors in order to cue readers to revise their predictions (across different levels of meaning-making) along a trajectory that configures the narrative overall^7^. Predictive processing thereby complements recent work on enculturation and cognitive literary theory by providing a theoretically and empirically plausible mechanistic account of the different components of the reading process.

It is important to note that the enculturated predictive processing account is markedly different from other accounts of reading and reading comprehension such as Johnson-Laird (1983), Kintsch (2004), and Zwaan and Radvansky (1998), which have influenced accounts of the relationship of mind-wandering and reading^8^. First, unlike these accounts, enculturated predictive processing seamlessly integrates perception, action, cognition, attention, and simulation, all of which are at the core of understanding reading, mind-wandering, and their relation. Second, while Johnson-Laird (1983), Kintsch (2004), and Zwaan and Radvansky (1998) remain largely neutral about the physical realization of the reading process. By contrast, enculturated predictive processing accounts for empirical findings that concern neuronal and ocular-motor processes that implementing processes of reading. Third, unlike mental models (Johnson-Laird, 1983) and situation models (Zwaan and Radvansky, 1998; Kintsch, 2004), the models at play in enculturated predictive processing are both generative and probabilistic, which does conceptual and empirical justice to the dynamicity and flexibility of the reading process. Finally, the concepts of “prediction” and “inference” in the context of enculturated predictive processing differ remarkably from the ways in which these concepts are used in other accounts of reading. Both “prediction” and “inference” refer to sub-personal probabilistic density distributions across time, rather than to cognitive processes ascribed to readers as a whole. For example, readers expect a noun after “ein prächtiger” on a personal level, but they usually do not think “where is the noun” unless Mann adds several additional qualifiers, delaying the completion of the syntactic segment. With these qualifications in place, we now proceed to show how enculturated predictive processing can contribute to a better understanding of reading and mind-wandering.

## A New Perspective on the Relation of Mind-Wandering and Reading

What does such a perspective on reading as an encultured practice that unfolds through loops of predictions and prediction errors now have to say about the studies on mind-wandering and reading? On a general level, it is striking that the studies on mind-wandering using reading as a control task make no allowance for different levels of reading skill in their participants and that they rarely make a distinction between different types of reading, ranging from skimming across the text to being absorbed in a narrative. Even if one assumed the simple relationship between mind-wandering and reading which we criticized in the State of research: Mind-wandering and reading, better reading skills would have an effect on mind-wandering. Contradictory hypotheses are possible on the basis of the arguments presented in these studies. Better readers might do less mind-wandering, if better reading skills correlated to higher attention or more control over tuning out. Better readers might do more mind-wandering, if better reading skills correlated to ease of processing. The literature we surveyed puts no hypotheses forward in this connection even though they arguably would affect the relationship between reading and mind-wandering which is the target of its investigations. Once we consider reading as an enculturated and predictive capacity that we master to different degrees and in different contexts, its relationship to mind-wandering gets much more complicated.

How would the perspective on reading as an encultured predictive practice allow us to conceptualize it in relation to mind-wandering? This is a question of epistemic active inferences on the part of the reader. Through palpable epistemic active inference, we actively manipulate the world so that it fits our predictions. Eye movements would be the most important example, but adjustments of body positions, e.g., how you sit in your reading chair, would also count as epistemic active inference in this case. Through virtual epistemic active inference, we generate virtual scenarios in which the world is modified in different ways to find out which manipulation works best (for example when you imagine rearranging your furniture to make space for a reading chair)^9^. Such virtual epistemic active inferences can be understood as “on-task” but nevertheless involving a decoupling from the immediate stimulus. This case clearly is qualitatively different from the case of ambiguity resolution, for example, where syntactically ambiguous words lead to longer reading times that are associated with processing difficulty (Fine et al., 2013). In contrast to mind-wandering, this case can be described as “on task” and coupled to the immediate linguistic context.

Literary texts can be considered to have a “design” on readers in the sense that they provide a carefully arranged sensory flow that can lead to a particular sequence of prediction errors (Kukkonen, 2016, Forthcoming a). The probability design of *Confessions of Felix Krull*, for example, invites readers to consider how a man whose existence defies social distinctions (he is a con-artist after all) nevertheless constantly configures his world through larger cosmological orders. Readers can run little alternative scenarios in which they explore the implications of the seemingly fixed social orders and Krull's seemingly smooth movement through them. When you read a narrative, you can for example create counterfactual scenarios in which the events might develop and thereby probe which predictions about the plot and overall coherence of narrative development seem to hold the greatest promise. Readers would then run their own alternative version(s) of the narrative which is modified along with the actual narrative when plot events occur. Again, these alternative versions would be “on-task” in terms of reading but depart from the immediate stimulus of the written word in front of readers in a way that for many experimental set-ups would be indistinguishable from mind-wandering.

A change in the embodied language of the text can provoke such virtual epistemic active inferences on the part of the reader, because the text shifts its precision (see Kukkonen, Forthcoming b for more details). We have seen an example with the shifting precision of “stehen” (“stand”) in the passage on Andromache that we discussed earlier. Precision, in the predictive processing model, is equivalent to attention. On a sub-personal level, our attention is directed to the prediction errors that are expected to be the most relevant. Eye-movements, understood as palpable epistemic active inference, indicate which words are re-evaluated in the course of a precision shift. Importantly, it is also conceivable that projections of counterfactual scenarios not rooted in individual words are associated with specific properties of eye movements. These properties would be clearly distinct from current findings on eye movement patterns that are exclusively concerned with the properties of eye movements targeted at previously determined critical words at a sentence level. If the probability design of a text indicates a shift in precision, for example through verbs that shift between the embodied and the abstract, this encourages readers to realize virtual epistemic active inferences to scope out the scenario that fits best with a coherent narrative. Here might be tilting points into tuning out, because, particularly in literary texts, it is up to the creativity of the readers how they spontaneously generate these virtual epistemic active inferences. However, epistemic active inference in general is modulated by precision estimates, which is equivalent to the direction of attention under predictive processing. This means that virtual epistemic active inference is rendered possible by the concurrent direction of attention. In these contexts, tuning-out should be considered as task-related, goal-directed and stimulus-dependent. These observations relate to attempts to capture the dynamics of mind-wandering, which have recently demonstrated that free-moving thought and task-relatedness are not mutually exclusive (Mills et al., 2018) and the fact that not all instances of what psychological research on mind-wandering calls “tuning out” are unproductive. In proficient and expert readers, this precision-modulated interplay of palpable and virtual epistemic inference would be guided by the probability design of the (literary) text.

Readers with different skills in reading and different degrees of exposure to literary texts will find it more or less difficult to engage in mind-wandering that predictive processing describes in terms of virtual epistemic active inferences. Better readers, then, are used to responding to subtle signals from the text that facilitate tracing sensory input that gives rise to the most precise prediction error. This theoretical frame allows us to sort out the conflicting hypotheses outlined above. Better readers do less mind-wandering of the zoning-out variety and more mind-wandering of the tuning-out variety. Because they are enculturated in developing virtual epistemic active inferences in response to precision shifts in texts, readers easily develop thoughts that move away from what the words on the page reference immediately. Because such epistemic active inferences are geared toward working out what will be precise prediction errors, that is, what it will be worth paying attention to, readers nevertheless engage in a mode of mind-wandering that is rather controlled and to the point. In this sense, the cases of mind-wandering of the “tuning out” variant that we are interested in would not be cases of “unguided attention” (Irving, 2016) and “unguided thinking” (Irving and Thompson, 2018). Since attention is equivalent to the estimation of precision, it would be guided by expectations about the relevance of prediction error signals. In turn, these precision estimates are informed by previous processes of prediction error minimization through perceptual and epistemic active inference. Furthermore, in many cases, “tuning out” while reading is not a process of “unguided thinking” either. The thinking process is guided by the reader's background knowledge (encoded in predictions) and the probability design of the text. This background knowledge is the result of enculturation and multiple reading experiences across time. On a sub-personal level, predictions steeped in background knowledge have a profound causal influence on the entire prediction error minimization process through perceptual inference, palpable epistemic active inference, and virtual epistemic active inference.

The enculturated predictive processing framework with its emphasis on the importance of probability designs provides us with a refined and more nuanced account of reading that helps specify the role of mind-wandering episodes in reading processes. In particular, our view on the relationship of reading and mind-wandering suggests that at least some cases of “tuning out” are an integral component of the reading process, and not an opposing force that prevents readers to actively engage with the text. This has important implications for future experimental research.

## Concluding Remarks and Outlook

Reading as a control task in the studies on mind-wandering reviewed in the State of research: Mind-wandering and reading needs to be reconsidered. It is not the simple, on-task activity as which it has been constructed in these studies, in particular when it comes to literary texts. As a cultural practice, reading is learnt and performed at different degrees of proficiency, and as carefully crafted artifacts, literary texts guide readers' attention in significant ways without completely controlling it. Reading itself, we have argued, feeds off mind-wandering and therefore cannot be posited as its opposite without further qualification. Enculturated predictive processing and the notion of probability designs in literary texts are one way to conceptualize how mind-wandering is shaped in the reading process and how the cultural skill of reading and the structure of the literary text create a different cognitive engagement than free-form mind-wandering. Readers, we have suggested, engage in epistemic active inferences that posit virtual scenarios (akin to tuning out) but that are part of a larger meaning-making process in anticipation of the designed prediction errors of the text's probability design. We propose not to dismiss studies that compare mind-wandering to reading but rather to specify the process of reading in relation to mind-wandering. Such a specification in dialogue with existing work can lead to more accurate and nuanced hypotheses to be tested in future experiments. Furthermore, it would be consistent with recent proposals to study mind-wandering within a family-resemblance framework^10^, which is based on the assumption that mind-wandering is a “fuzzy-boundaried and heterogeneous construct” (Seli et al., 2018, p. 485) and to attend to the temporal dynamics of mind-wandering (Christoff et al., 2016; Mills et al., 2018).

Our discussion of how literary reading has been treated in at least a sub-set of studies on mind-wandering has already pointed toward important factors that could contribute to the onset, type (i.e., tuning out or zoning out), and content of mind-wandering episodes during reading. In what follows, we consider from the perspective of enculturated predictive processing how the influence of these factors could be integrated into the future empirical study of mind-wandering to enrich our understanding of mind-wandering, reading, and their delicate relationship.

The literary text is not an indifferent stimulus for “reading.” Experimenters should consider the stylistic or narratorial features of texts they choose and whether they increase the likelihood of the onset of a mind-wandering episode. Can they be described as “precision shifts” in terms of shifts between interoception and exteroception or between concrete and imagined embodiment? If such mind-wandering leads to virtual epistemic active inferences, it might also show in eye-movement patterns. For example, there is empirical evidence that eye movement patterns associated with the visual imagination of a certain scene is strikingly similar to the eye movement patterns associated with the actual perception of that scene in the here and now (Laeng and Teodorescu, 2002). It is a theoretical possibility that the eye movement patterns that have been associated with mind-wandering could actually be ocular-motor signatures of the imagination of a fictional scenario guided by the probability design of the text.

A further question that could be investigated is how the difference between “tuning out” and “zoning out” manifests itself in differences in their realization in terms of virtual epistemic active inference. Is there a difference in the quality of readers' mind-wandering when characters mind-wander, too? The idea is that these cases of *second-order mind-wandering* are characterized by a specific qualitative profile, where second-order mind-wandering episodes can be defined as readers' engagement with the mind-wandering episodes of a fictional character.

We have suggested above that there are texts that require some degree of mind-wandering for successful processes of reading. If this suggestion is warranted, then a limited sub-category of mind-wandering in reading can be classified as being “on task,” depending on the probability design of the text. An important consequence of this would be that the texts deployed in mind-wandering studies need to be analyzed from this point of view and experimental results need to be reconsidered.

In any event, we submit that the properties of the text materials used in empirical studies on mind-wandering promise to have a direct impact on the quantitative and qualitative properties of mind-wandering episodes. This is consistent with the recent empirical finding that the properties of literary texts have an important impact on the reading experience (Moore and Schwitzgebel, 2018). For this reason, we suggest that future research should develop selection criteria for text material to increase the explanatory value of the interpretation of empirical data. In addition to narrative strategies and style, this also concerns the length of the text that is presented to readers and the paratextual elements that guide attention, such as chapter headings and chapter breaks. Furthermore, the actual reading habits and the devices on which the texts are presented should be taken into account. Are these texts presented on a screen? Do readers usually read literary texts on screen or in paper books? Substantial differences between these surrogates have been discussed in empirical reading studies (Mangen and Kuiken, 2014). Are people practiced in reading literature? The last two questions are targeted at the participants, whose habits and skill levels should be carefully detected by using standard print exposure measures (Stanovich and West, 1989; Moore and Gordon, 2015). Furthermore, recent empirical evidence suggests that there are inter-individual differences in the reported reading experiences (Moore and Schwitzgebel, 2018). This might also have implications for the future empirical exploration of the links between mind-wandering and reading.

It would be imperative to study longer reading durations to ascertain what happens to mind-wandering in reading for, say, an hour. Does it increase overall mind-wandering? Is there a shift between different kinds of mind-wandering? What happens to mind-wandering on a second reading? If texts present an interface for “scaffolded learning” (Clark, 1997; Sterelny, 2012), then mind-wandering should not exclusively occur as a phenomenon of attention fatigue (at least within a reasonable time span of reading), because the text supports and guides attention in productive ways. Experienced readers should be capable of reading without distractions for much longer than inexperienced readers. Such factors of reading practices could confound results from the mind-wandering and reading studies.

These deliberations outline that (1) greater attention needs to be paid to the text and the readers in studies on mind-wandering, that (2) foundational studies on the role of mind-wandering in literary reading are necessary and that (3) these might lead to a re-evaluation of existing studies on mind-wandering. Approaching mind-wandering from a perspective informed by literary study and enculturated predictive processing might allow for more helpful distinctions between different kinds of mind-wandering. In a further step, then, mind-wandering could be reconnected to other activities that are associated with significant activations in the default mode network such as processes of personal memory, future thinking, or creative cognition.

## Author Contributions

All authors listed have made a substantial, direct and intellectual contribution to the work in equal terms, and approved it for publication.

### Conflict of Interest Statement

The authors declare that the research was conducted in the absence of any commercial or financial relationships that could be construed as a potential conflict of interest.

## Footnotes

^1^The authors have made direct and substantial contributions to this work in equal terms.

^2^More recently, Mills et al. (2018) have suggested to add “freely moving thought” as an additional dimension that can help specify the properties of mind-wandering episodes under experimental conditions.

^3^Methodologically speaking, research on mind-wandering, just as research on other classes of conscious experience, can be described as an attempt to systematically relate subjective reports from a *first-person perspective* to objective scientific considerations from a *third-person perspective* (Metzinger, 2003, 2004, 2017). The ultimate goal of this naturalistic approach is to describe and explain first-person phenomena in terms of scientific, third-person perspective accounts. In what follows, our main strategy is to provide a theoretical account of the relationship between mind-wandering in reading from a third-person perspective in order to develop a scientific understanding of the reported subjective phenomenal experiences. Self-reports in the cases at hand can provide an understanding of the subjective experiences of people participating in the studies reviewed and discussed in the main text. However, we would like to note that these first-person perspective reports are evaluated and interpreted from a scientific third-person perspective.

^4^Interestingly, Seli et al. (2017) suggest that meta-awareness and intentionality are distinct dimensions that can become dissociated as mind-wandering episodes unfold. For present purposes, however, we will focus on the meta-awareness dimension without denying that it will be an important task to further determine the relationship between meta-awareness and intentionality in future experimental and theoretical research.

^5^As already mentioned above in the main text, there are some recent notable exceptions to the trend to neglect the lexical properties of texts in studies that specifically examine the relationship between mind wandering and reading. These exceptions include the determination of word frequencies (Feng et al., 2013), the measurement of text complexity in terms of the Kincaid Grade Level scale (Forrin et al., 2017; Mills et al., 2017), which assesses the difficulty of a text relative to grade levels at school, and the specification of the concreteness of the words in the reading materials. Thanks to a reviewer for bringing this to our attention.

^6^This and the following translations from the German are ours.

^7^This account also applies to the case of reading expository texts, since they will have a structure of argument if not necessarily a structure of narrative and since both literary and expository texts presuppose background knowledge on the part of readers. At least one notable exception needs to be considered though: Literary and expository texts have different lexical properties and, on many occasions, this difference would also have an impact on the reading process as it can be described in terms of enculturated predictive processing. For the purpose of this paper, we confine ourselves to literary reading. More generally, since predictive processing unites perception, action, cognition, simulation, and attention and has been applied to a whole range of phenomena (see Clark, 2016 for an overview), there is no reason to assume that there are principled differences between a predictive processing style description of reading literary and expository texts. We are grateful to a reviewer for pressing us on this point.

^8^Thanks to a reviewer for suggestion to be explicate the relation between enculturated predictive processing and other accounts of reading.

^9^Unless otherwise indicated, in this section we focus on this sub-class of epistemic active inference, which is associated with the generation of virtual scenarios in the sense of Pezzulo et al. (2016).

^10^This family-resemblance framework allows for the differentiation of natural kinds of mind-wandering that share similarities *and* phenomenological and functional differences. From the perspective of Wittgenstein (2010) family resemblances, our account would suggest that, in certain constellations, skilful reading is a member of the mind-wandering family in the sense specified above.
